# Unveiling the dynamic effects of major depressive disorder and its rTMS interventions through energy landscape analysis

**DOI:** 10.3389/fnins.2025.1444999

**Published:** 2025-03-05

**Authors:** Chun-Wang Su, Yurui Tang, Nai-Long Tang, Nian Liu, Jing-Wen Li, Shun Qi, Hua-Ning Wang, Zi-Gang Huang

**Affiliations:** ^1^School of Life Science and Technology, Xi'an Jiaotong University, Xi'an, Shaanxi, China; ^2^Research Center for Brain−inspired Intelligence, Xi'an Jiaotong University, Xi'an, Shaanxi, China; ^3^Department of Psychiatry, First Affiliated Hospital of Air Force Medical University, Xi'an, Shaanxi, China; ^4^Department of Psychiatry, The 907th Hospital of the PLA Joint Logistics Support Force, Nanping, Fujian, China; ^5^Department of Psychiatry, The 904th Hospital of the PLA Joint Logistics Support Force, Changzhou, Jiangsu, China

**Keywords:** major depressive disorder (MDD), repetitive transcranial magnetic stimulation (rTMS), fMRI, resting state dynamics, energy landscape

## Abstract

**Introduction:**

Brain dynamics offer a more direct insight into brain function than network structure, providing a profound understanding of dysregulation and control mechanisms within intricate brain systems. This study investigates the dynamics of functional brain networks in major depressive disorder (MDD) patients to decipher the mechanisms underlying brain dysfunction during depression and assess the impact of repetitive transcranial magnetic stimulation (rTMS) intervention.

**Methods:**

We employed energy landscape analysis of functional magnetic resonance imaging (fMRI) data to examine the dynamics of functional brain networks in MDD patients. The analysis focused on key dynamical indicators of the default mode network (DMN), the salience network (SN), and the central execution network (CEN). The effects of rTMS intervention on these networks were also evaluated.

**Results:**

Our findings revealed notable dynamical alterations in the pDMN, the vDMN, and the aSN, suggesting their potential as diagnostic and therapeutic markers. Particularly striking was the altered activity observed in the dDMN in the MDD group, indicative of patterns associated with depressive rumination. Notably, rTMS intervention partially reverses the identified dynamical alterations.

**Discussion:**

Our results shed light on the intrinsic dysfunction mechanisms of MDD from a dynamic standpoint and highlight the effects of rTMS intervention. The identified alterations in brain network dynamics provide promising analytical markers for the diagnosis and treatment of MDD. Future studies should further explore the clinical applications of these markers and the comprehensive dynamical effects of rTMS intervention.

## 1 Introduction

Major Depressive Disorder (MDD) is a prevalent human brain dysfunction, leading to symptoms such as depressed mood, anhedonia, cognitive dysfunction, and increased suicidal tendencies (Wiebenga et al., 2022). It significantly impairs psychosocial functioning and diminishes individuals' overall well-being (Malhi and Mann, 2018). However, the specific etiology and pathogenesis of MDD remain unclear, and there is an urgent need to explore the diagnosis of depression and effective treatment. Despite the efficacy of antidepressants, approximately one-third to one-half of individuals with MDD do not experience a full response to multiple antidepressant treatments, and others may only achieve partial relief (Rush et al., 2009; Cipriani et al., 2018). There is a clinical need for more effective antidepressant treatments. Repetitive Transcranial Magnetic Stimulation (rTMS) is a noninvasive brain stimulation technique and its efficacy in the treatment of depression has been demonstrated by several original studies and meta-analyses (Luber et al., 2017; Tik et al., 2023a; Valiengo et al., 2022; Chen et al., 2017; Slotema et al., 2010). However, the mechanisms underlying rTMS intervention in MDD are still not fully understood, which limited further improvements of this promising technique. Therefore, the brain science basis and effects of rTMS intervention become hot topics in MDD study (Anand et al., 2005; Drevets et al., 2008; Hasanzadeh et al., 2019; Dai et al., 2019; Kaster et al., 2023). In such studies, the functional magnetic resonance imaging (fMRI) serves as an essential technical tool which supports basic measurements of the activities of brain regions (region of interest, ROI) and function-specific brain networks (Galioulline et al., 2023; Tik et al., 2023b; Kim et al., 2023).

In 2011, Menon (Menon, 2011) proposed the “triple network model” (TNM) theory. It is demonstrated that altered functional connectivity of the default mode network (DMN), the central execution network (CEN), and the salience network (SN) is closely related to various mental illnesses, including depression (Chibaatar et al., 2023). It was found that task engagement or exposure to external stimuli markedly diminishes DMN activity while concurrently activating the CEN (Bressler and Menon, 2010). The SN is believed to dynamically regulate the activities of the DMN and CEN (Sridharan et al., 2008). During the past decades, the TNM has been increasingly utilized in the investigation of MDD (Supekar et al., 2019; Zheng et al., 2015).

On the other hand, an increasing number of research studies have begun to investigate brain function and its disorders from the perspective of dynamics (Deco et al., 2011; Parkes et al., 2022; Zhou et al., 2023, 2024). Specifically, inspired by thermodynamics and statistical physics, researchers have embarked on efforts to quantify and intuitively investigate the global dynamics and transition properties of the brain's complex systems (Ye et al., 2023). This shift toward a physics-informed perspective has fostered novel analytical methods and enriched the understanding of brain dynamics. Several studies have employed statistical mechanics-related approaches to explore the brain. For instance, Breakspear and Stam (2005) proposed a multiscale neural framework using nonlinear oscillators and wavelet decomposition to explore neural dynamics across different organizational scales. Deco and Jirsa (2012) introduced the concepts of criticality and multistability, using methods from statistical physics to simulate and analyze the dynamic behavior of brain cortical networks. Chialvo (2010) explained the self-organizing behavior of the nervous system and the emergence of complex dynamic patterns based on complex systems theory. These studies provide new perspectives and methods for neuroscience research and the treatment of neurological disorders. Among these novel approaches for investigating brain dynamics, the energy landscape analysis (Ezaki et al., 2017; Ashourvan et al., 2017) based on spin glass theory (Binder and Young, 1986) drew a great deal of attention since its robustness, easy training and elegant pictures. Energy landscape analysis is a data-driven method based on statistical mechanics that is used to investigate states and transitions in complex systems. In the study of resting-state brain dynamics, researchers employ energy landscape analysis to describe the energy distribution and stability of the brain network in different states (Watanabe and Rees, 2017). The definitions of state, energy, and stability are similar to those in the Ising model in statistical physics. The microstates of a system are configurations of the binary states of the brain regions it contains (akin to spins), and each configuration corresponds to a state function similar to the energy of a spin system in the Ising model, based on its probability of occurrence. However, in this energy function, both the spin coupling strength and the field strength coefficients are heterogeneous and need to be fitted from the observed data. This state function (i.e., energy) indicates the stability of the system's microstates, with lower energy states indicate greater stability. The coarse-grained state of the system is a combination of local minima in the energy landscape of microstates and all microstates within their basins. This analytical approach can reveal dynamic features of the resting-state brain, such as state stability and transition probabilities. Through energy landscape analysis, researchers can gain a deep understanding of the resting-state brain activities and their modulations. For example, it has been found that the locus coeruleus-norepinephrine (LC-NE) system can modulate the switch of the triple network activities (He et al., 2023), and the energy landscape analysis provides a novel dynamical perspective to investigate such modulations (Munn et al., 2021).

In this paper, we focus on exploring the dynamic characteristics of MDD by delineating the temporal and state-dependent activity patterns of the brain, as well as the governing principles of state transitions. By contrasting these dynamics with those of healthy individuals, we seek to identify functional anomalies that may contribute to the pathogenesis of MDD. Moreover, we will investigate the intervention effects of rTMS on MDD by comparing the corresponding pre- and post-treatment dynamics. Our aim is to elucidate the specific mechanisms by which rTMS modulates brain function, thereby providing theoretical underpinnings and practical guidance for MDD therapy.

## 2 Materials and methods

### 2.1 Data and participants

The data used in this study are from Xijing Hospital, including three subject groups: pre-intervention major depressive disorder patients (MDD_Prior), post-intervention MDD patients (MDD_Post), and healthy controls (HC). It comprises 38, 38, and 34 participants, respectively. All participants met the following inclusion criteria: (1) right-handed, (2) aged 18–60, (3) HAMD-17 >17, (4) non-psychotic, (5) negative urine screen and pregnancy test for females, and (6) no TMS/MRI contraindications.

The MRI-navigated rTMS treatment was delivered by a Black Dolphin Navigation Robot system (SmarPhin 760, Solide Brain Control Medical Technology Co., Ltd., Xi'an, China), targeting a specific position in the left dorsolateral prefrontal cortex (DLPFC) which exhibits the strongest negative correlation with the subgenual anterior cingulate cortex (sgACC) (Fox et al., 2012) based on functional negative correlation with resting-state fMRI. The individualized rTMS stimulation target is defined as the peak subunit on the DLPFC that is most negatively connected to the sgACC according to Cole et al. (2022). However, the definition of the sgACC was slightly different from that in Cole et al. (2022). In the current study, No. 187 and 188 atlases based on Brainnetome Atlas (BNA) (https://atlas.brainnetome.org/bnatlas.html) (Fan et al., 2016) were selected as the sgACC to improve the signal-to-noise ratio and avoid mixing information comes from the corpus callosum. After the definition of the individualized stimulation target, 5-day sgACC FC-guided rTMS treatment, i.e., SAINT, was administered to each patient (Cole et al., 2020, 2022). Participants received a daily regimen of 6 iTBS sessions, each characterized by a structured protocol of 20 iTBS cycles. Within each cycle, three magnetic pulses were delivered at 0.2-s intervals, resulting in a total of 600 pulses per session. The clusters of pulses were separated by 8-s intervals, ensuring optimal temporal dynamics for neuromodulation. The stimulation intensity was carefully calibrated to 90% of the individual's motor threshold, ensuring both safety and efficacy. Each session was spaced by 30-min intervals to allow for physiological recovery and integration of the treatment effects. Over the course of five consecutive days, this intervention resulted in a cumulative total of 3,600 pulses per day, amounting to 18,000 pulses over the treatment period. HAMD-17 scores were collected before and after treatment, coinciding with fMRI. Before rTMS intervention, the HAMD-17 scores of MDD patients were 28 ± 4.52. After rTMS intervention, the HAMD scores of MDD patients were 9.52 ± 6.01, exhibiting a significant difference (*p* < 0.001). This difference indicates rTMS efficacy, providing a clinical foundation for exploring its mechanism.

High-resolution MRI data were acquired using a 3.0 T uMR 780 scanner before and after treatment. During resting-state fMRI, participants kept their eyes closed, maintaining wakefulness while avoiding deliberate thinking. The parameters for resting-state fMRI were as follows: slices = 35, repetition time = 2,000 ms, echo time = 30 ms, slice thickness = 4 mm, matrix size = 64 × 64, field of view = 224 mm × 224 mm, flip angle = 90°. The resting-state fMRI sessions lasted ~12 min. The post-treatment MRI acquisition was conducted 1–2 days after the TMS intervention.

We conducted our research with rigorous adherence to ethical guidelines following approval from the Medical Ethics Committee of Xijing Hospital (Approval No. KY20202066-X-1). Our study was executed in strict accordance with the approved clinical research protocol, adhering to the principles of Good Clinical Practice (GCP), ensuring that informed consent was obtained from all participants. We diligently safeguarded the rights, privacy, and safety of our subjects, while maintaining full compliance with relevant national and international regulations, thereby contributing to the scientific community with a study of integrity and ethical excellence.

### 2.2 Preprocessing

The preprocessing of resting-state functional magnetic resonance imaging (fMRI) data primarily utilized the software tools SPM12 (http://www.fil.ion.ucl.ac.uk/spm/) and Gretna. The main objectives of preprocessing were threefold, (1) to minimize errors introduced during data acquisition or due to physiological characteristics of the brain, (2) to test the statistical assumptions of the model and transform the data to satisfy these assumptions, (3) to standardize the brain region locations across different subjects for subsequent group analyses, thereby enhancing the validity and sensitivity of such analyses. The preprocessing steps typically included fMRI data visualization, censoring, ICA-denoising, temporal alignment, head motion correction, spatial smoothing, linear trend removal, band-pass filtering, structural-functional alignment, and the removal of white matter and cerebrospinal fluid signals.

Subjects with displacement greater than 2 mm, head motion exceeding 2 degrees, or those receiving sham stimulation were excluded, resulting in a final participant count for each group in this study: 28 patients with MDD (age range: 18–54 years, 22 females, mean age: 27.35 ± 9.73 years) and 29 healthy controls (HC) (age range: 22–38 years, 13 females, mean age: 30.90 ± 4.59 years).

The main brain regions selected in this study are the DMN, CEN, and SN, using Region of Interest (ROI) templates obtained from the Stanford University NeuroImage and Neuroinformatics Laboratory (Shirer et al., 2012). This ROI template was used to extract the time series of different brain regions within these three networks in the low-frequency oscillation (LFO) frequency band. The template divides the DMN, CEN, and SN into 22, 12, and 19 ROIs, respectively. However, not all ROIs from the template were included in this study. Some ROIs are either too small or too deep, making it challenging to extract their activity signals. Therefore, to ensure data reliability, we excluded such ROIs. Specifically, 22 ROIs from the DMN, eight from the CEN, and 15 from the SN were selected. In the context of functional magnetic resonance imaging, the finite length of time series data, which typically spans several hundred to a thousand time points, imposes a constraint on the dimensionality of the system's microstates. Specifically, the number of ROIs that can be effectively included in the analysis is limited, as exceeding this limit may lead to a pronounced bias in the estimation of state occupancy probabilities and transition probabilities due to data scarcity. The upper bound of the dimension *N*_*m*_ can be empirically determined by the approximation that $2^{N_{m}}$ is roughly equivalent to the product of the data length and the number of subjects under investigation. This relationship serves as a pragmatic guideline for ROI selection in fMRI studies to ensure the reliability and validity of the subsequent statistical inferences. The greater the number of ROIs in the model, the higher the potential number of configurations. If the amount of data is insufficient, such as when the time series is too short, some configurations may not be observed. This inevitably leads to biases in estimating configuration probabilities. Due to the dimension limitations of energy landscape, the three networks were further divided into six subnetworks: dorsal DMN (dDMN), posterior DMN (pDMN), ventral DMN (vDMN), anterior SN (aSN), posterior SN (pSN), and CEN. The division of subnetworks follows the approach of the Stanford University NeuroImage and Neuroinformatics Laboratory (Shirer et al., 2012). Corresponding brain regions, Brodmann areas (BA), and Montreal Neurological Institute (MNI) standard space coordinates for these six networks are detailed in Tables 1–3 in Appendix 1).

### 2.3 Energy landscape analysis

Energy landscape analysis (Watanabe et al., 2014) is an approach that investigates the interactions between localized brain regions from the perspective of statistical physics. The concept of “energy” is operationalized by selecting specific state variable functions, describing the energy associated with different brain regions, thus composing the brain system states. These states are essentially modeled based on empirical distribution data derived from fMRI data of various brain regions. By employing the model defined by the energy function, the energy landscape of the brain system within the state space, also referred to as the energy terrain of the system, can be derived. The configuration of the energy landscape mirrors the stability and interactions of the brain states, and it can elucidate a series of the system's dynamic characteristics. The key points of energy landscape analysis involve fitting a pairwise maximum entropy model (MEM) (Watanabe et al., 2013; Ezaki et al., 2018) based on the BOLD signals and characterizing the metastable dynamics based on the energy landscape of the brain states. Given the extensive data required for this method, this study aggregated fMRI signals from subjects within the same group before conducting the pairwise MEM fitting.

For each network, there exist *N* ROIs, named by *i* (*i* = 1, 2, …, *N*). For each ROI of each subject, a threshold was set based on the average signal value over the entire time length. If the signal value exceeded this threshold, the ROI was considered active. The BOLD signals of each ROI were binarized based on the threshold. At each time point, the state of a single ROI *i* is σ_*i*_ = 0,1 (*i* = 1, 2, …, *N*) and the state of a network consisting of *N* ROIs will be represented by an *N*-dimensional binary vector **σ** = [σ_1_, σ_2_, …, σ_*N*_] which indicates the network's activity pattern. There are 2^*N*^ possible activation patterns for each network. For each activity pattern **σ**, its probability of occurrence was calculated using Equation 1, where *n*_**σ**_ is the occurrence frequency of the state in the time series, and *T* is the length of the time series.


(1) $$\begin{array}{l} p\left(\sigma \right)=\frac{n_{\sigma }}{T}. \end{array}$$

The “energy” associated with each activity pattern **σ** was calculated using Equation 2 as follows


(2) $$\begin{array}{l} E\left(\sigma |J,h\right)=-\frac{1}{2}\underset{i\neq j}{\sum }J_{ij}\sigma_{i}\sigma_{j}-\underset{i}{\sum }h_{i}\sigma_{i}. \end{array}$$

In the case of MEM, the occurrence frequency *p*(**σ**) of each activity pattern **σ** follows the Boltzmann distribution as shown in Equation 3.


(3) $$\begin{array}{l} p\left(\sigma |J,h\right)=\frac{\exp-E\left(\sigma |J,h\right)}{\sum_{\sigma^{\prime }}\exp E\left(\sigma^{\prime }|J,h\right)}, \end{array}$$

where *J*_*ij*_ and *h*_*i*_ are parameters representing the strength of interaction between ROIs *i* and *j* and the trend of isolated activation of ROI *i*, respectively. In this definition, a lower energy value corresponds to a higher occurrence frequency of an activity pattern over time.

**Figure 1:**
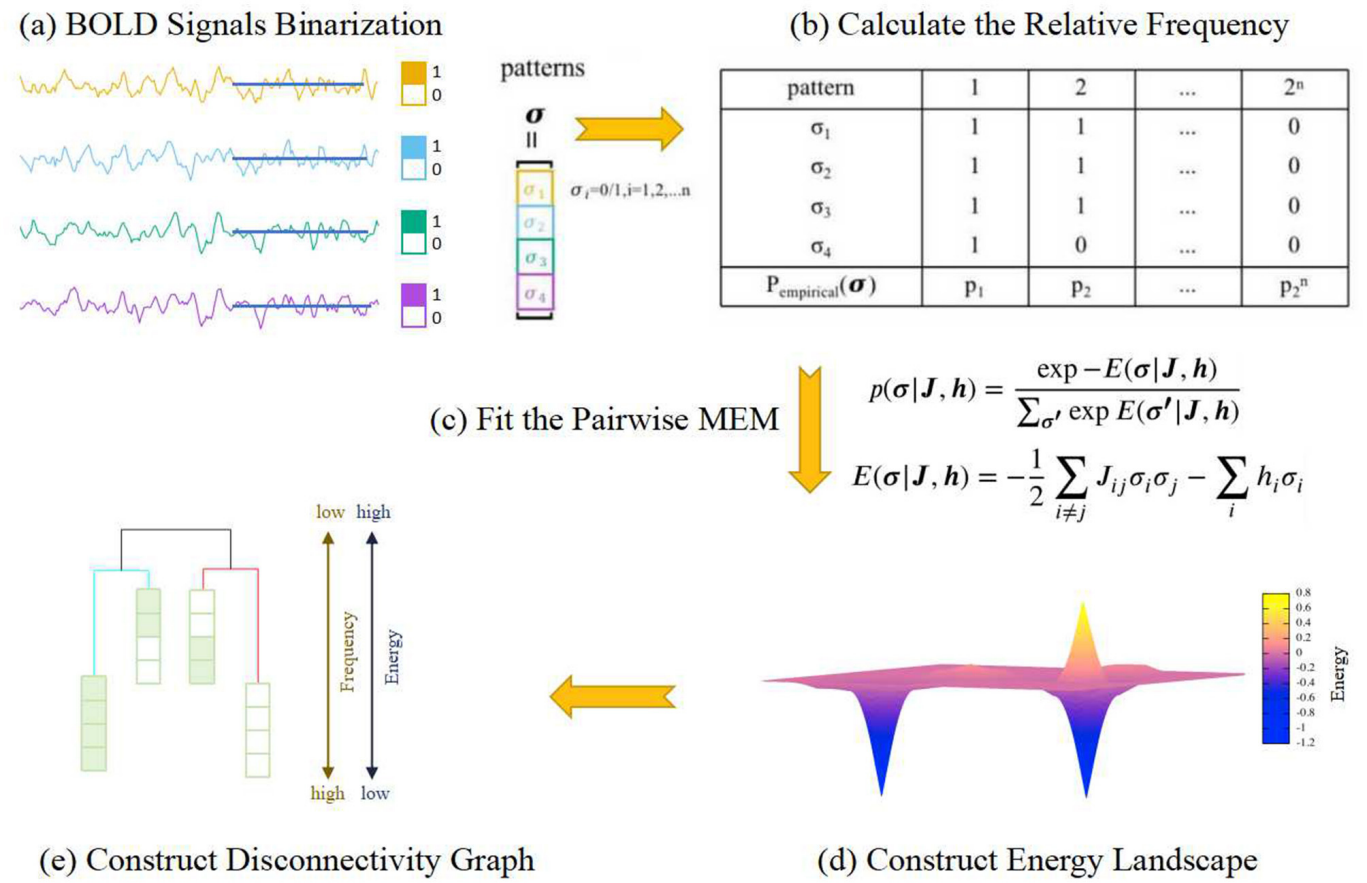
The flowchart of the energy landscape modeling. **(A)** The BOLD signal for each ROI is binarized based on its temporal mean. At each time point, if its value is higher than the mean, it is assigned a value of 1; otherwise, it is assigned a value of 0. Thus, the state of a single ROI will be similar to a spin in Ising picture. **(B)** The empirical probability of each pattern **σ**, configuration of the spins, is estimated according to the time series. **(C)** A function called “energy function,” which maps each pattern **σ** to a potential, is fitted under the constraint of the empirical probabilities of the patterns. **(D)** The energy landscape is obtained based on the energy function. **(E)** From the energy landscape, the attractors and their basins, along with the barriers between different basins, can be identified and then the disconnectivity graph can be constructed. Each attractor and its basin form a metastate. In this paper, whenever we mention the brain states while analyzing brain dynamics, we are referring to the metastates.

MEM is a method based on gradient descent that maximizes the entropy of the Boltzmann distribution. The parameters ***J*** ∈ ℝ^*N*×*N*^ and ***h*** ∈ ℝ^*N*^ are iteratively updated to enhance the accuracy of the model fitting, ultimately leading to an optimized model. Based on this optimized model, the energy landscape of the system can be constructed. The dynamic characteristics of different brain states can be reflected in the energy landscape, including local minima, energy basins, disconnectivity graphs, and energy barriers. The process of energy landscape analysis is illustrated in Figure 1.

Within the energy landscape, among the constructed 2^*N*^ activity patterns (also known as state vectors), two states differing by only one element were considered adjacent states. Thus, each state corresponds to *n* adjacent states. If the energy value of a state is lower than that of all its *n* adjacent states, the state is defined as a local minimum state, corresponding to the lowest point of a basin in the energy landscape.

Disconnectivity graphs can be used to characterize the primary features of the dynamics, including the energy levels of local minima, metastates, and the energy barriers between adjacent basins. A disconnectivity graph is a tree-like branching structure, where different branches represent different local minima. The height of the branches corresponding to adjacent local minima reflects the energy barrier between them. Compared to the energy landscape, the disconnectivity graph provides a more concise and intuitive representation of the relationships between various states. The specific steps for constructing the disconnectivity graph are as follows:

1. Create a superlattice composed of state vectors, where each state is connected to its adjacent states.
2. Set an energy threshold *E*_*th*_ as the maximum energy among all current states.
3. Remove all states with energy *E* ≥ *E*_*th*_ and their corresponding connections.
4. Verify that at least one path connects each local minimum state in the superlattice.
5. Repeat steps 3 and 4, setting *E*_*th*_ as the maximum remaining energy value, until all local minima are mutually disconnected.

Record the *E*_*th*_ value set when two local minima are first disconnected, as it represents the potential energy barrier between the two basins. This process results in the construction of the disconnectivity graph for local minima.

Energy landscape analysis is a computational approach that offers an intuitive interpretation of multivariate time series data. In summary, the analysis involves four main steps: (1) data binarization, (2) estimation of the pairwise maximum entropy model (Boltzmann distribution), (3) construction of disconnectivity graphs and energy basins for local minima, and (4) calculation of dynamic metrics for the energy landscape. Empirically, the energy landscape analysis method is most effective when the number of variables ranges from ~6 to 15. For a greater number of variables, the computational cost becomes substantial and interpreting the results becomes challenging, while fewer variables may result in poor fitting accuracy and stability.

### 2.4 Statistical analysis

In this study, independent-sample t-tests were used to analyze the statistical significance of differences between groups for various parameters. This method determines whether there are significant differences in the population means of two independent samples. The threshold for statistical significance was set at *p*_0_ = 0.05, with *p*-value below this threshold indicating a significant difference. Moreover, since multiple comparative tests were conducted on the same dataset, a Bonferroni correction was applied to mitigate the risk of type I errors due to multiple testing.

Additionally, to ascertain whether changes in dynamic indicators of MDD, identified based on differences between pre-intervention MDD patients and the healthy control group, were related to rTMS intervention, correlations with clinical scales were calculated. A *p*-value < 0.05 was considered indicative of a significant correlation. The strength of the correlation is denoted by the magnitude of the correlation coefficient: values above 0.7 suggest a very strong relationship, values between 0.4 and 0.7 indicate a moderate relationship, and values between 0.2 and 0.4 suggest a weak relationship. Even if the correlation coefficient is below 0.2, a significant *p*-value implies a weak yet existent correlation.

## 3 Results

### 3.1 Changes of metastable dynamics induced by MDD suffering and rTMS intervention

Based on the energy models derived from energy landscape analysis (see Supplemental Materials for details), we have identified that certain local minima are more prevalent. It is crucial to understand that these energy values do not correspond to any biological form of energy; rather, they are statistical metrics representing the likelihood of each brain activity pattern occurring over time. We posit that patterns with lower energy values are more likely to manifest and tend to be more stable.

**Figure 2:**
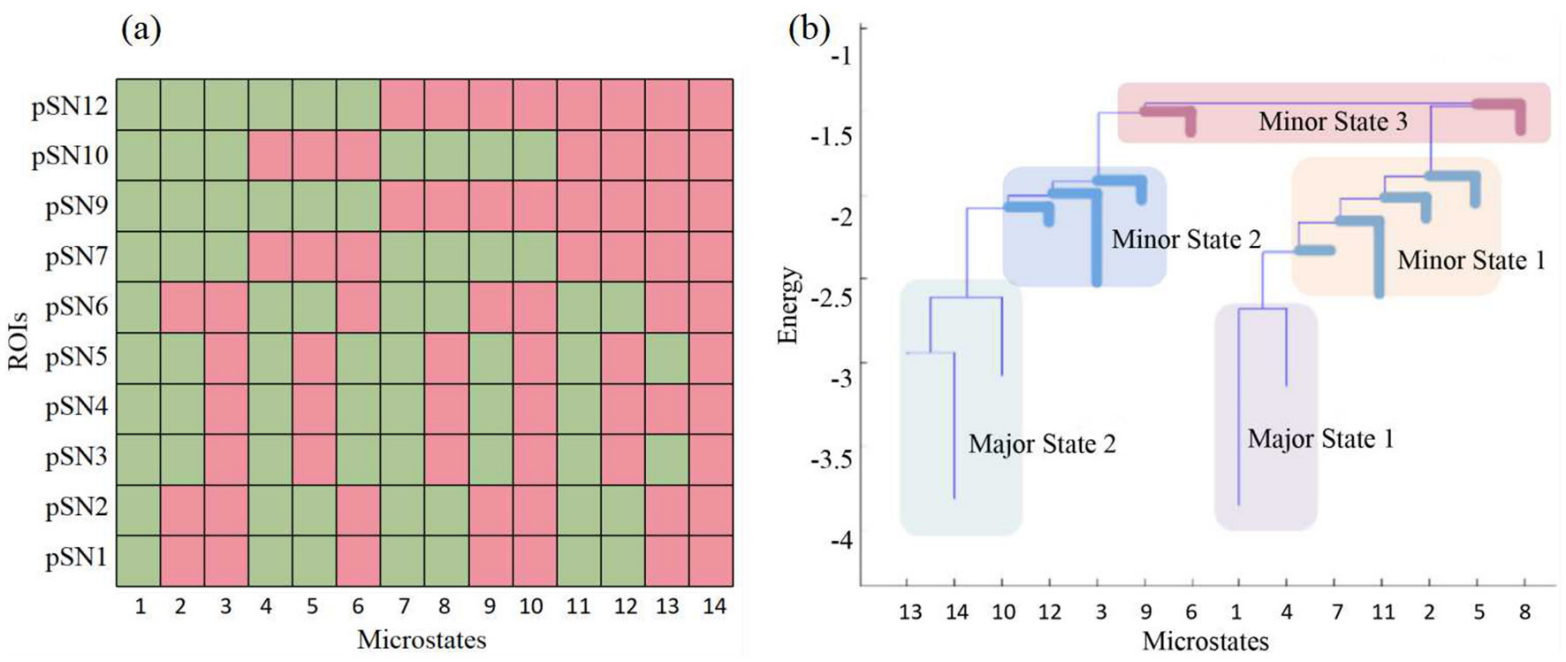
Major states, minor states and the activity patters of the microstates. **(A)** The activity patters of the 14 attractors of pSN dynamics in HC group. **(B)** The major states and minor states of pSN in HC group. The pSN from the HC group is utilized as an illustrative example in this context due to its possession of the greatest number of attractors and minor states. In **(A)**, green indicates inactive, while red indicates active.

Consequently, we have delineated two primary brain state groups, designated as major state 1 and major state 2. These groups typically encompass a fully inactive state and a fully active state, respectively, along with their adjacent states. All other states are classified as minor state 1. However, if any states within minor state 1 exhibit consistent intergroup characteristics, such as significantly higher energy, they are categorized as minor state 2 or even minor state 3 (Figure 2). For meaningful intergroup comparisons, we ensure that the same patterns are indexed consistently across all participant groups. That is, state groups are uniform and representative for all three groups of participants. Any inconsistent patterns are relegated to minor state 1.

Through energy landscape analysis, we obtained disconnectivity graphs and activity patterns for six brain networks, averaged across the three groups. To discern markers useful for evaluating MDD and monitoring rTMS efficacy, we consider three dynamic characteristics. To apply the Bonferroni correction, we first determine the total number of indicators under statistical scrutiny. For the six brain networks, the number of indicators varies due to the division of states, contingent on the number of state groups. The formula for the number of indicators *N*(indicators) is given by


(4) $$\begin{array}{l} N\left(\text{indicators}\right)=2\times n\left(\text{state groups}\right)+n\left(\text{transitions}\right). \end{array}$$

The equation for *n*(transitions) is


(5) $$\begin{array}{l} \{\begin{array}{c} 2,n\left(\text{state groups}\right)>2\\ 1,n\left(\text{state groups}\right)=2 \end{array} \end{array}$$

For the Bonferroni correction, the total number of comparisons *M* equals *N*(indicators). Therefore, the significance level α is adjusted to 0.05/M, and this Bonferroni-corrected *p*-value is used to identify the indicators. After applying the Bonferroni correction, we pinpoint several network features that meet the correction criteria, which may serve as potential indicators for distinguishing MDD and monitoring the effects of rTMS.

Our analysis reveals distinct alterations in the dynamic brain states in the MDD group and that after rTMS intervention. Significant differences are found in the frequency and duration of brain states when comparing HC and MDD, as well as pre- and post-intervention conditions across all subject groups. These differences highlight the potential of these dynamic characteristics as biomarkers for MDD progression and intervention efficacy.

**Figure 3:**
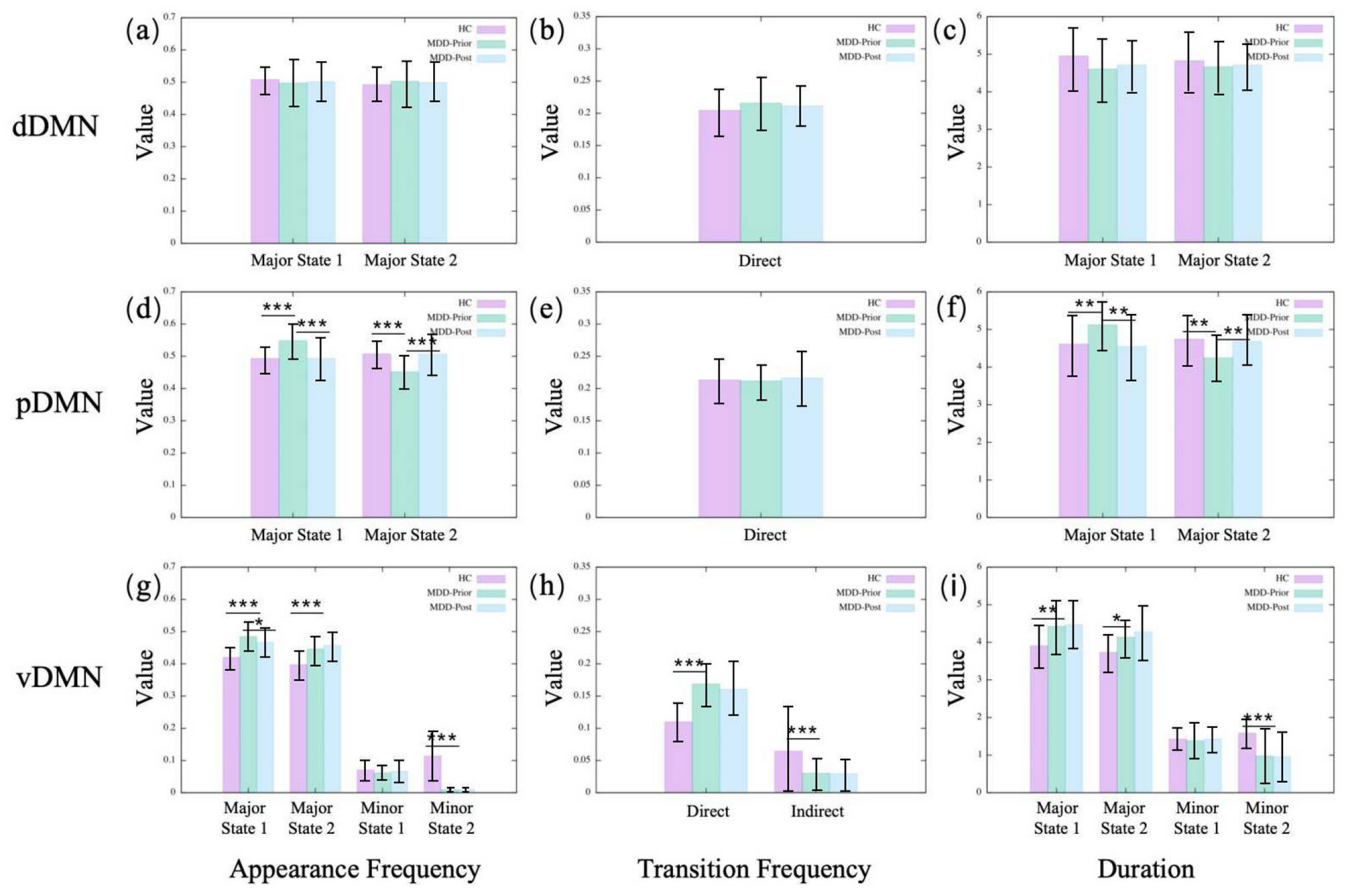
The statistics of dynamical characters of subnetworks in DMN for the three groups. **(A, D, G)** illustrate the appearance frequencies of the merged metastates of dDMN, pDMN and vDMN, respectively. Metastates with low potential energy and large basins are termed major states, while those with high potential energy and small basins are referred to as minor states. **(B, E, H)** illustrate the transition frequencies of the merged metastates of dDMN, pDMN and vDMN, respectively. The direct transitions between major states are referred to as direct transition frequencies, whereas the transitions between major states that are mediated through minor states are referred to as indirect transition frequencies. **(C, F, I)** illustrate the durations, i.e., lifetime, of the merged metastates of dDMN, pDMN and vDMN, respectively. The duration of a state refers to the length of time for which that state persists. HC, MDD-Prior, and MDD-Post refer to the healthy control group, the group of MDD patients before rTMS intervention, and the group of MDD patients after rTMS intervention, respectively. In statistical analyses, asterisks are employed to signify the levels of statistical significance: a single asterisk (*) denotes a *p*-value < 0.05, a double asterisk (**) indicates a *p*-value < 0.01, and a triple asterisk (***) corresponds to a *p*-value below 0.001. These symbols are used consistently in all subsequent figures.

**Figure 4:**
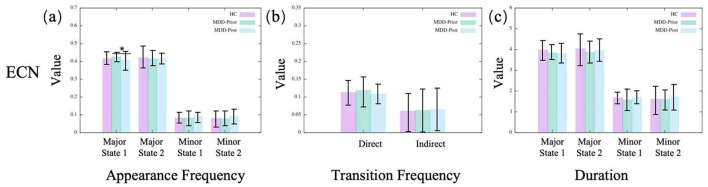
The statistics of dynamical characters of CEN for the three groups. **(A–C)** illustrate, respectively, the appearance frequencies, transition frequencies, and durations of the merged metastates.

In the dDMN, we observe no significant alterations for MDD patients compared to the HC group (Figure 3). A similar result is found in the CEN, as shown in Figure 4. Moreover, neither dDMN nor CEN is significantly affected by the rTMS intervention. However, in all other subnetworks of the TNM, MDD and rTMS intervention may induce changes in some dynamic indicators.

#### 3.1.1 Brain state dynamics alterations in MDD patients

**Figure 5:**
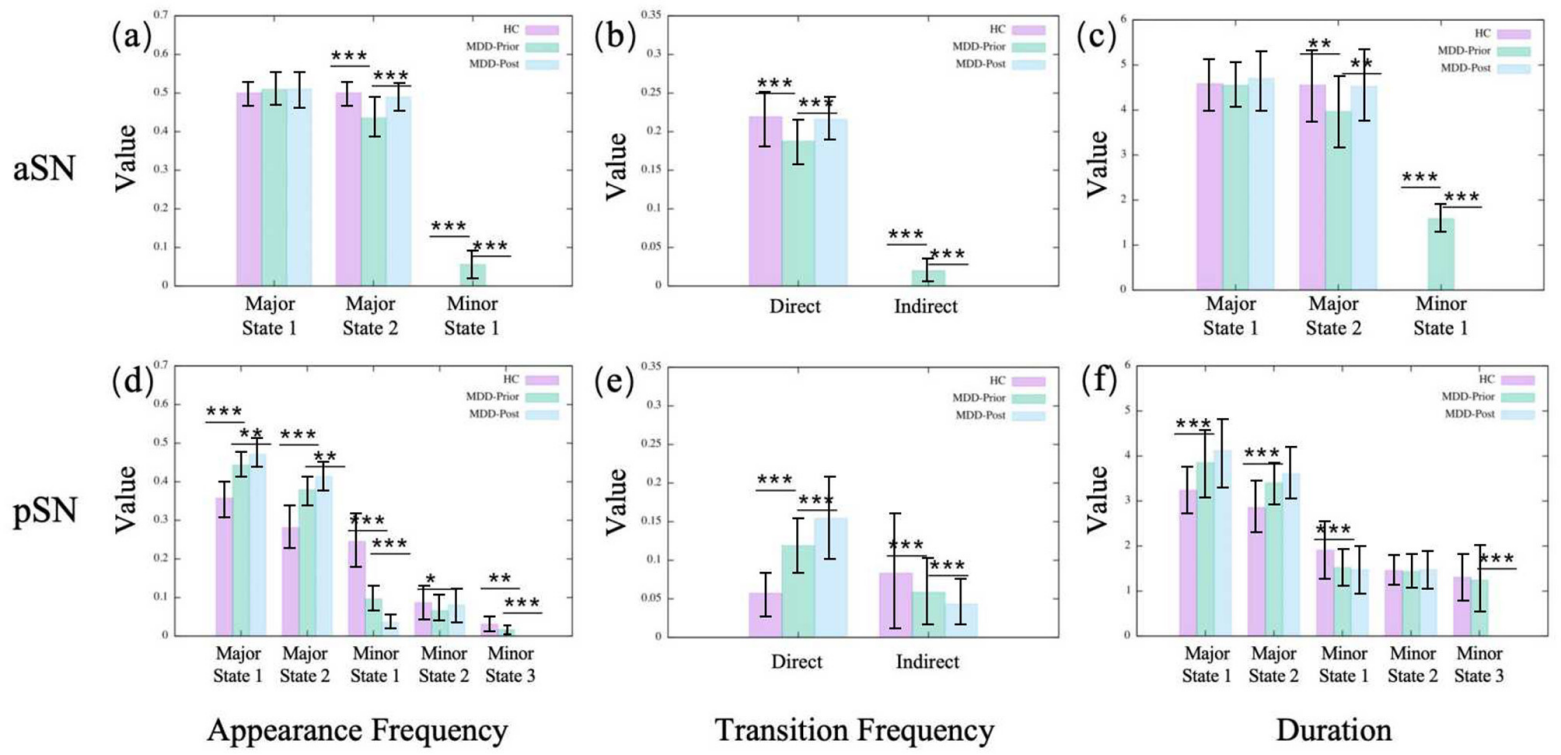
The statistics of dynamical characters of subnetworks in SN for the three groups. **(A, D)** illustrate the appearance frequencies of the merged metastates of aSN and pSN, respectively. **(B, E)** illustrate the transition frequencies of the merged metastates of aSN and pSN, respectively. **(C, F)** illustrate the durations of the merged metastates of aSN and pSN, respectively.

In the pDMN, the major state 1 becomes more prominent in the MDD group, compared to the HC group, with increases in both occurrence frequency and average duration, while major state 2 shows a reduction in these indicators (Figure 3). The vDMN shows an overall increase in the activity of major states in the MDD group. Both the occurrence frequencies and average durations of the two major states significantly increase. Conversely, the minor states of the vDMN show a decrease across all indicators, suggesting a less frequent occurrence of this state in the MDD group. In addition, the direct transitions between the major states increase, while the indirect transitions decrease (Figure 3). The indirect transitions are that transitions between the major states mediated by minor states. In the SN, the aSN shows that for the MDD group, the occurrence frequency and average duration of major state 2 decrease while major state 1 remains unchanged. The occurrence frequency and average duration of the minor states increase, the direct transition frequency significantly decreases, and the indirect transition frequency increases (Figure 5). The results of the pSN indicate that for the MDD group, both the occurrence frequency and average duration of the major states 1 and 2 significantly increase, while all minor states show a decrease in occurrence frequency and average duration. This suggests transitions from minor states to major states, implying enhanced dominance of the major states. Additionally, the increased direct transition frequency between major states further confirms this point (Figure 5).

#### 3.1.2 Alterations in brain state dynamics induced by rTMS intervention

The observed alterations within the pDMN in the context of MDD are reversible following rTMS intervention. Specifically, in the pDMN, the major state 2 emerges with enhanced salience following the intervention, with increases in both occurrence frequency and average duration. Conversely, major state 1 of the pDMN exhibits a significant decline in the aforementioned metrics. However, in the vDMN, almost all alterations in MDD remain unchanged after rTMS intervention, except for the appearance frequency of the major state 1. The phenomena within the aSN exhibit intriguing and distinct characteristics. The alterations in the aSN are completely reversed by rTMS intervention, since all the dynamic indicators are pulled back to the HC levels. After rTMS, the occurrence frequency and average duration of major state 2 in the aSN increase, while those of the minor states decrease. The direct transition frequency increases and the indirect transition frequency decreases, coinciding with the disappearance of minor state 1. This indicates a reassertion of the dominance of major states, especially major state 2. However, in the pSN, the alterations are not reversed. Conversely, all alterations that occur in the MDD brain continue to be reinforced. It is found that after rTMS, the occurrence frequency and average duration of the major states in the pSN of the MDD brain are further increased, while those of the minor states are further decreased. Additionally, the increased direct transition frequency between major states is further increased. The results indicate that rTMS intervention amplifies the variabilities observed in the pSN within the MDD group, potentially constituting a limitation that prevents the current rTMS intervention program from achieving the desired therapeutic outcome for MDD.

#### 3.1.3 Correlation of potential dynamic indicators with clinical pathologies

To elucidate the link between the amelioration of dynamic brain indicators and rTMS intervention, we conducted a correlation analysis with clinical scale scores. The aim is to determine whether alterations in the brain's dynamic indicators parallel improvements in depressive symptoms.

In this study, a total of 10 scales were considered, with the details presented in Table 4 in Appendix 2. These scales assess behavioral outcomes at various time points during the treatment period. Scales 1–6 provided measurements at baseline, as well as after 5, 15, and 30 days of treatment. Scales 7–9 provided insights at two time points: baseline and after 5 days. Scale 10, known as HAMD-6, was assessed daily from the initial baseline up to 5 days following the intervention. This chronological evaluation displays a consistent pattern of decreasing scores, suggesting the potential effectiveness of rTMS intervention. Correlation coefficients were computed to examine the relationship between the scores on these scales and the dynamic indicators of brain activity. Positive coefficients denote a concurrent decrease in both scale scores and indicator values, while negative coefficients indicate an inverse relationship.

**Figure 6:**
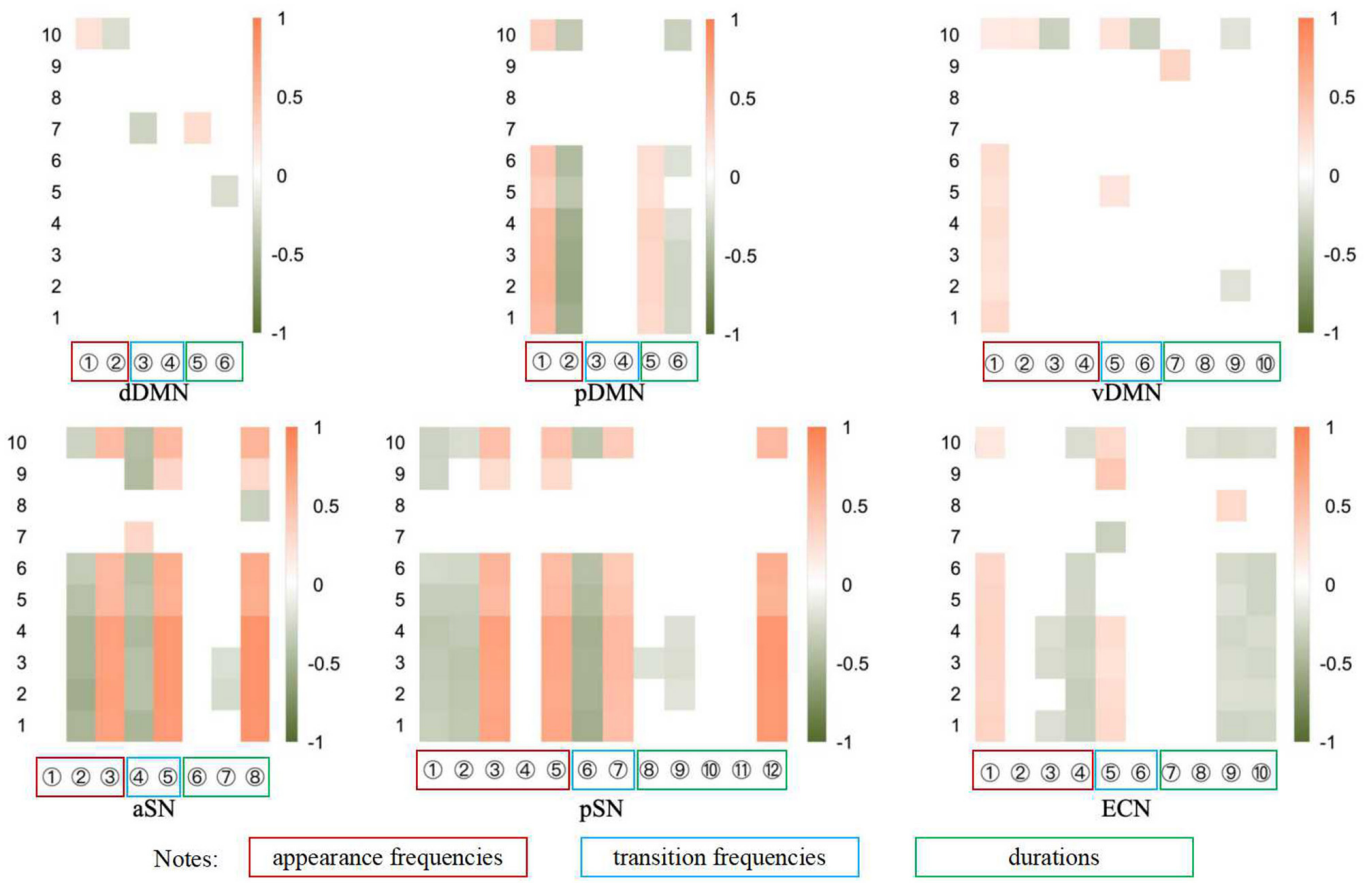
The heatmap of correlations between the potential dynamical indicators and the 10 clinical scales (see Supplemental Materials for details). The vertical numbers represent clinical scales as described in Table 4 in Appendix 2, while the horizontal numbers represent dynamical indicators. Each subnetwork encompasses *n* (*n* ≤ 5) states which belong to the 5 state groups. The labels on the horizontal axis sequentially represent the occurrence, i.e. the appearance frequency, of the *i*th state (*i* = 1, 2, … , *n*), the direct transition frequency, the indirect transition frequency, and the duration of the *i*th state (*i* = 1, 2, … , *n*). The 5 state groups and the states of different subnetworks are shown in Table 5 in Appendix 3. Only the correlations that pass the significance test are displayed in color.

The correlation analysis results depicted in Figure 6 suggest relative consistency across Scales 1–6, with Scale 10's outcomes aligning with those of Scales 1–6 in most instances. However, the results for Scales 7–9 appear less consistent, which we attribute to the fact that only two time points are insufficient to establish a trend or even a correlation. In this paper, we primarily focus on the findings pertaining to Scales 1–6. Figure 6 presents the correlation coefficients that reached statistical significance condition *p* < 0.05, with the horizontal axis representing the indicators and the vertical axis denoting the scale number. The color coding within the figure, orange for positive and green for negative, indicates the strength of the correlation, with deeper hues signifying a stronger correlation. Given that higher scores on depression scales denote more severe depressive symptoms, a positive correlation coefficient implies that an increase of the indicator value indicates a negative therapeutic effect, whereas a negative coefficient suggests a positive therapeutic effect.

Most of these observations are consistent with the findings from brain state dynamics analyses. Specifically, metrics that show a significant increase following the intervention exhibit a negative correlation with scale scores, whereas a significant decrease is associated with a positive correlation. This suggests that the changes in most indicators are clinically significant. However, despite many significant changes in the vDMN, no significant consistency is observed in the consistency analysis with clinical practice except for the appearance frequency of major state 1. In addition, although we only find a significant change in the appearance frequency of major state 1 in the CEN, many indicators (especially those of the minor state) show consistency with the scale results.

### 3.2 Underlying temporal relation changes revealed by metastates transitions

Based on the initial energy landscape analysis, which focuses on the dynamics of single subnetwork for the identification of dynamic indicators pertinent to diagnostics and therapy, the investigation does not encompass the dynamic interplay between different subnetworks. To address this gap, the present study expands the scope to include an examination of the inter-network dynamic relationships by scrutinizing the frequency of state transitions within networks in relation to specific transition relationships, herein defined as the “motivate rate” (MR).

Within each network, *n* distinct state groups are identified, resulting in *m* potential state transition relationships. For each specified transition relationship, designated as “a,” the frequency with which “a” occurs in network A immediately preceding or in concurrence with its manifestation in network B is computed, denoted as *N*_a,*A* → *B*_. Concurrently, the aggregate frequency of “a” across each network is tallied, denoted as *N*_a,*A*_. These data points facilitate the computation of the motivate rate.

The motivate rate from network A to network B is defined as


(6) $$\begin{array}{l} MR\left(\text{A}\to \text{B}\right)=\frac{\sum_{m}N_{\text{a},\text{A}\to \text{B}}}{\sum_{m}N_{\text{a},\text{B}}}, \end{array}$$

where *m* = *n*!/(*n* − 2)!.

To ascertain the dynamic interplay between networks A and B, we compare the motivate rate from A to B, *MR*(*A* → *B*), with that from B to A, *MR*(*B* → *A*). If *MR*(*A* → *B*) exceeds *MR*(*A* → *B*), it is inferred that network A precedes network B in their dynamic relationship.

**Figure 7:**
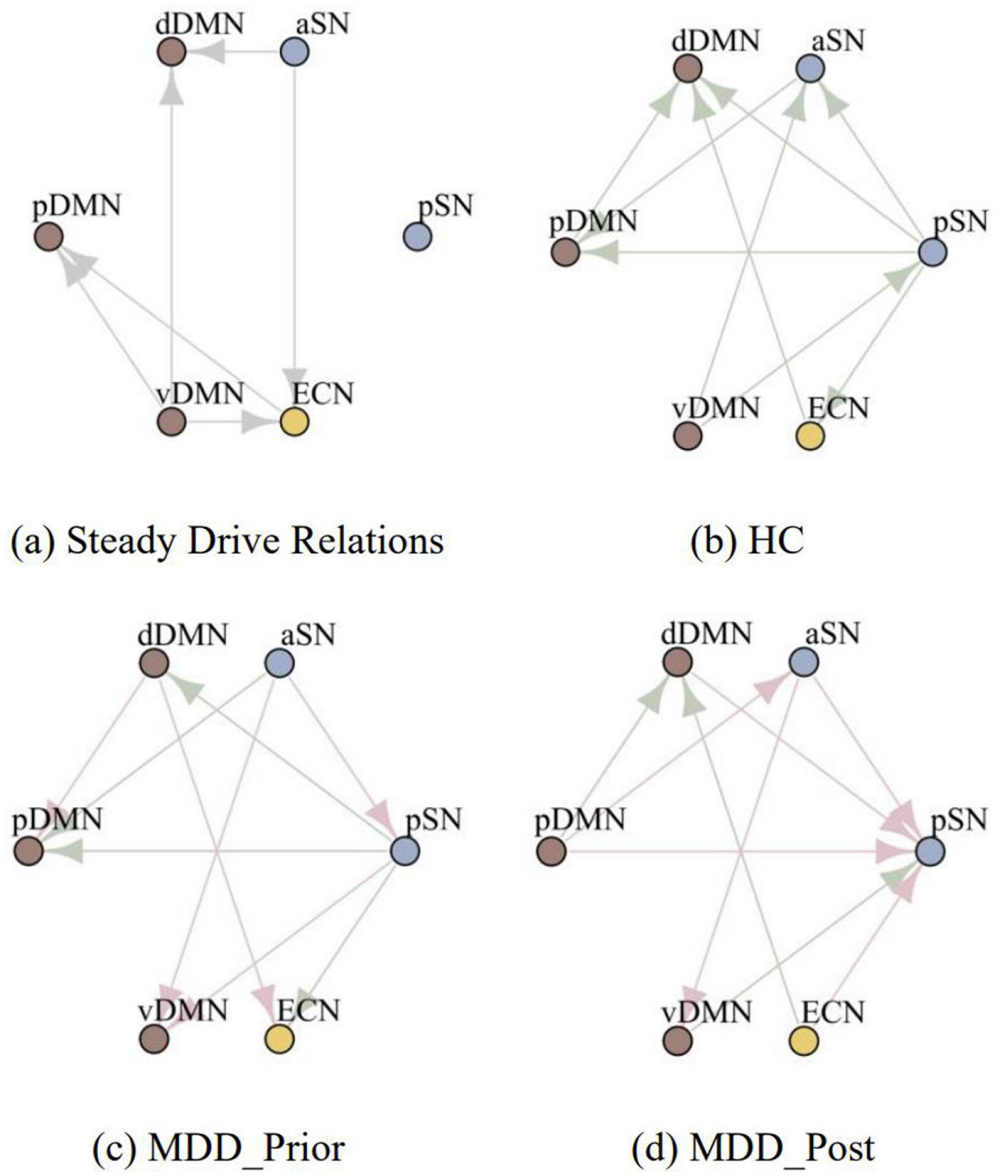
The temporal relations among the six subnetworks for different groups. **(A)** The temporal relationships that consistently exist among the three groups. **(B–D)** depict the temporal relationships among subnetworks for the healthy control group, the MDD patients prior to rTMS intervention, and the MDD patients following rTMS intervention, respectively, with the steady temporal relations omitted.

Since the length of individual participants' BOLD signals is too short, precluding accurate estimation of the motivated rate, we eschewed the calculation of temporal relationships for individual subjects and the subsequent statistical testing. Instead, we aggregated data at the group level and calculated the temporal relationships between subnetworks for each group, facilitating comparative analysis across distinct groups. By calculating the motivate rate, we first identified several temporal relationships that consistently exist among the three groups of subjects, as shown in Figure 7A. These temporal relationships may not be associated with MDD, and thus we do not include them in the subsequent inter-group comparisons and thus we do not include them in the subsequent inter-group comparisons (Figures 7B–D).

Comparing the temporal relationships between participants in the HC group and the MDD_Prior group, we found changes in five pairs of temporal relationships. There are two temporal relationships related to the dDMN, where the state transitions of the dDMN undergo a notable transformation, shifting from a mode of posterior occurrence to one of predominant anterior occurrence; two temporal relationships related to the vDMN, where the vDMN changes from the anterior occurrence mode to the posterior occurrence mode; Additionally, there are two temporal relationships related to the aSN, where the aSN changes from the posterior occurrence mode to the anterior occurrence mode.

Comparing the temporal relationships between participants in the MDD_Prior group and the MDD_Post group, we found changes in seven pairs of temporal relationships, with the two pathological temporal relationships related to the aSN remaining unchanged.

## 4 Discussion

In addition to the changes in metastable dynamics, the major focus of this study is on major state 1 and major state 2, which are associated with the fully deactivated state and fully activated state, respectively. The fully activated state may indicate functional integration and coordination of multiple brain regions. When multiple brain regions are simultaneously activated, they may participate in complex cognitive, emotional, or control processes involving information exchange and integration across multiple brain networks. In contrast, the fully deactivated state may indicate functional segregation or a lack of coordination among these brain regions.

It is suggested that self-reference and emotion are relevant factors in sustaining or evoking activity of default mode network (Gusnard et al., 2001; Harrison et al., 2008). Additionally, it is proved that the role of DMN for MDD patients in self-referential processing is impaired (Sheline et al., 2009; Nejad et al., 2013). Our results further corroborate this point, as the pDMN and vDMN both show increasing activity in major state 1 in the MDD group. In other words, MDD patients are more often in a fully deactivated state, indicating lower efficiency in executing cognitive tasks.

Among the three groups of participants, significant differences are observed in the pDMN, vDMN, aSN, and pSN networks. Since results with significant differences are the most convincing, we focus only on indicators with significant differences, especially those that have consistently significant correlations with clinical scale results. To facilitate the interpretation of the combined effects of multiple indicators, we provide the following explanations. If a state group has a high occurrence frequency and long duration, it is considered to have a greater functional role in brain activity in that region. This suggests that such a state group is stable and potentially critical for the sustained functioning of that region. However, if a state group has a high occurrence frequency and short duration, it is considered significant in the transitional activities in that region. This implies that the state group is involved in quick shifts or changes in brain activity, rather than sustained processes.

For the changes related to MDD, the pDMN and pSN networks exhibit a significant increase in the frequency and duration of their major states, indicating an elevated functional role within these regions. Conversely, the dDMN and aSN show changes suggesting a nuanced shift toward states with transitional significance, with major state 2 in the dDMN and major state 1 in the aSN becoming more prominent in their respective networks' transitional dynamics. Regarding the effects post-intervention, there is a discernible enhancement in the functional roles of major state 1 in the dDMN and major state 2 in the pDMN, as well as both major states in the aSN, reflecting the intervention's impact on these networks. The pSN's increase in all state indicators and the CEN's rise in major state 1 alongside more frequent indirect transitions suggest a complex reconfiguration of functional and transitional roles within these networks, highlighting the intervention's broad modulatory effects.

In our investigation of the pDMN, vDMN, and aSN, we identified several indicators that not only undergo significant changes in MDD group but also demonstrate substantial correlations with scale scores. These indicators may serve as reliable diagnostic criteria for MDD. Within the pDMN, the frequency and average duration of major state 1 are considered reliable indicators. In the vDMN, the frequency and average duration of major state 2, along with the frequency of major state 1, stand out. For the aSN, the direct transition frequency of major states and the negative correlation of the indirect transition frequency indicate their diagnostic potential.

According to the scale results, patients with MDD received effective treatment after rTMS intervention. Based on the changes in temporal relationships we found that three out of five pathological temporal relationships are effectively restored after rTMS intervention. We speculate that the three pairs of temporal relationships related to the dDMN and vDMN are associated with brain region activities related to MDD pathophysiology. The unchanged dominant temporal relationship status of the aSN after rTMS intervention may indicate that the effects of rTMS on the aSN are limited. Moreover, it appears that the effect of rTMS intervention is reflected in the reversal of temporal relationships in brain activities, but it does not precisely identify pathological temporal relationships, instead performing a generalized reversal. This provides a new perspective for researching the precise mechanisms of rTMS in the treatment of depression.

Rumination is believed to be a critical characteristic in understanding how depression emerges and endures over time (Smith and Alloy, 2009). Evidence suggests that increasing levels of DMN dominance in depression are associated with higher levels of maladaptive, depressive rumination and lower levels of adaptive, reflective rumination (Hamilton et al., 2011). This excessive DMN activity is associated with the hippocampus and leads depression patients to excessively focus on negative memories in their autobiographical recollections (Young et al., 2016). In this study, the hippocampus is part of the dDMN network, and it is found that the dDMN shows altered activity in MDD group. This is reflected in the dDMN transitioning from a network preceded by the CEN and pDMN to one that precedes the CEN and pDMN. This finding aligns with the phenomenon of depressive rumination. Additionally, our research results further narrow the association between the DMN and rumination to the dDMN. Moreover, among the two altered temporal relationships observed in the vDMN, one pair exhibits the same aberrant temporal relationship, leading us to speculate that the vDMN also exerts a certain degree of influence on the phenomenon of rumination.

In our study, a key aspect of our methodology involved subdividing the CEN, SN, and DMN into sub-networks. This approach was necessitated by the dimension limitations inherent to energy landscape analysis, as detailed in our methods section. However, it is important to address potential concerns regarding how such subdivision, as well as the selection of specific brain regions, might influence the robustness and validity of our results. Indeed, energy landscape analysis is designed to capture the dynamic interactions and stability properties of the brain networks based on the selected ROIs. When we subdivide a larger network into smaller sub-networks, we focus on more localized and specific aspects of the overall network dynamics. While this may limit direct comparability to analyses conducted on the entire network without subdivision, it allows us to gain deeper insights into the specific sub-networks that are most relevant to the pathophysiology of MDD and the effects of rTMS. In this sense, the subdivision can be seen as a necessary trade-off between comprehensiveness and analytical tractability, rather than a source of bias. Furthermore, energy landscape analysis inherently involves a probabilistic and dynamic framework that can accommodate some degree of variability in the input data. The state transitions and stability properties that we examined are derived from the collective behavior of the selected ROIs, rather than relying on the specific characteristics of individual regions. This probabilistic nature of the analysis provides a certain level of robustness against minor perturbations in the selection of brain regions.

## 5 Limitations

This study, while providing significant insights into the brain's metastable dynamics and the effects of rTMS intervention in MDD, has several limitations to consider. Firstly, the focus on major and minor states may not encapsulate the full complexity of intermediate states. The study also does not provide a detailed mechanistic explanation for rTMS's therapeutic effects. Secondly, the sample size and demographic scope may limit the generalizability of findings. The proposed diagnostic criteria from the pDMN, vDMN, and aSN require further clinical validation. Lastly, uncontrolled confounding factors may affect the results, indicating the need for larger, more diverse samples and longitudinal studies to better understand MDD's pathophysiology and the nuanced effects of rTMS interventions.

## 6 Conclusions and future work

In conclusion, this study enhances our understanding of MDD by pinpointing novel dynamic indicators of the resting state networks (RSNs) in the human brain based on the energy landscape perspective, providing new potential biomarkers for diagnosis and treatment monitoring. The observed functional changes in these networks, especially the pathophysiology alterations in major state 1 and its subsequent recovery following rTMS, emphasize the clinical utility of these indicators. Furthermore, the reversal of most temporal relationships after rTMS intervention, including those in the dDMN that reflect rumination phenomena, provides new insights into the therapeutic mechanisms of rTMS.

Although our research sets the stage for future inquiries, it also underscores the inherent limitations of studies constrained by limited datasets and sample sizes. Future research should aim to validate these indicators in a broader demographic and enhancing analytical models to encapsulate the subtle spectrum of brain dynamics. Such initiatives could catalyze the development of personalized treatments, potentially increasing the effectiveness of interventions like rTMS for individuals with MDD.

Our work serves as a foundation for a deeper, more refined understanding of MDD and signals a possible paradigm shift in clinical practices for diagnosis and treatment. The quest to decipher the complexities of brain function in depression forges ahead, fostering the aspiration to improve the prognosis for those impacted by this condition.

## Data Availability

The raw data supporting the conclusions of this article will be made available by the authors, without undue reservation.
