# Supplementary material for: Unveiling the dynamic effects of major depressive disorder and its rTMS interventions through energy landscape analysis
Source: Front Neurosci. 2025 Mar 5;19:1444999. doi: 10.3389/fnins.2025.1444999 (PMC11920141; doi:10.3389/fnins.2025.1444999)
Supplement: Supplementary file 1 [file Presentation_1.pdf]

Appendix

Chun-Wang Su [1,2], Yurui Tang [1], Nai-Long Tang [3,4], Nian Liu [3,5], Jing-Wen Li [3], Shun Qi [2], Hua-Ning Wang [3,*], Zi-Gang Huang [1,2,*]

[1] *School of Life Science and Technology, Xi'an Jiaotong University, No.28, Xianning West Road, 710049, Xi'an, Shaanxi, China*
[2] *Research Center for Brain−inspired Intelligence, Xi'an Jiaotong University, No.28, Xianning West Road, 710049, Xi'an, Shaanxi, China*
[3] *Department of Psychiatry, First Affiliated Hospital of Air Force Medical University, No.127, Changle West Road, 710032, Xi'an, Shaanxi, China*
[4] *Department of Psychiatry, the 907th Hospital of the PLA Joint Logistics Support Force, 353000, Nanping, Fujian, China*
[5] *Department of Psychiatry, the 904th Hospital of the PLA Joint Logistics Support Force, 213000, Changzhou, Jiangsu, China*

Correspondence*:
xskzhu@fmmu.edu.cn, huangzg@xjtu.edu.cn

1 THE ROIS IN DMN, SN AND CEN

The detailed information of ROIs in DMN, SN and CEN is listed in the Tables 1, 2 and 3, respectively. This information includes names of brain regions (ROI), labels, left or right hemisphere, Brodmann area and MNI spatial coordinates.

Table 1. Names, abbreviation labels, and MNI spatial coordinates of ROIs in DMN.

Label	ROI	L/R	BA	X	Y	Z
dDMN_1	medial frontal gyrus	L	9	0	49	12
dDMN_2	angular gyrus	L	39	-48	-73	32
dDMN_3	superior frontal gyrus	R	6	18	38	51
dDMN_4	dorsal posterior cingulate gyrus	L	31	0	-57	30
dDMN_5	ventral anterior cingulate gyrus	R	24	0	-17	35
dDMN_6	angular gyrus	R	39	48	-66	29
dDMN_7	thalamus	L	-	-6	-6	3
dDMN_8	parahippocampal gyrus	L	36	-24	-37	-9
dDMN_9	parahippocampal gyrus	R	36	24	-21	-23
pDMN_1	ventral posterior cingulate gyrus	L	23	0	-35	28
pDMN_2	precuneus	R	7	0	-76	38
pDMN_3	inferior parietal lobule	L	40	-39	-64	46
pDMN_4	inferior parietal lobule	R	40	39	-64	46
vDMN_1	posterior cingulate	L	31	-12	-62	10
vDMN_2	middle frontal gyrus	L	10	-27	-6	59
vDMN_3	culmen	L	37	-30	-39	-20
vDMN_4	superior occipital gyrus	L	19	-36	-88	28
vDMN_5	posterior cingulate gurus	R	31	15	-56	13
vDMN_6	precuneus	L	7	-6	-61	56
vDMN_7	middle frontal gyrus	R	10	24	26	47
vDMN_8	culmen	R	37	27	-33	-23
vDMN_9	angular gyrus	R	39	43	-79	28

Table 2. Names, abbreviation labels, and MNI spatial coordinates of ROIs in SN.

Label	ROI	L/R	BA	X	Y	Z
aSN_1	middle frontal gyrus	L	10	-32	45	26
aSN_2	anterior insula cortex	L	13	-41	15	-2
aSN_3	cingulate gyrus	L	32	-2	17	45
aSN_4	middle frontal gyrus	R	10	28	43	26
aSN_5	anterior insula cortex	R	13	44	13	1
pSN_1	middle frontal gyrus	L	10	-40	36	35
pSN_2	inferior parietal lobule	L	40	-60	-38	35
pSN_3	precuneus	L	7	-9	-56	64
pSN_4	cingulate gyrus	R	31	11	-29	44
pSN_5	precuneus	R	7	11	-53	63
pSN_6	inferior parietal lobule	R	40	59	-32	31
pSN_7	thalamus	L	-	-12	0	-21
pSN_9	posterior insula cortex	L	13	-40	-16	-3
pSN_10	thalamus	R	-	11	-16	9
pSN_12	posterior insula cortex	R	13	39	-14	-7

Table 3. Names, abbreviation labels, and MNI spatial coordinates of ROIs in CEN.

Label	ROI	L/R	BA	X	Y	Z
LCEN_1	dosolateral prefrontal cortex	L	9	-35	21	53
LCEN_2	middle frontal gyrus	L	10	-44	46	-1
LCEN_3	inferior parietal lobule	L	40	-44	-65	44
LCEN_4	middle temporal gyrus	L	21	-65	-38	-12
RCEN_1	dosolateral prefrontal cortex	R	9	32	26	44
RCEN_2	middle frontal gyrus	R	10	35	62	7
RCEN_3	inferior parietal lobule	R	40	46	-54	49
RCEN_4	middle frontal gyrus	R	8	3	36	44

2 THE CLINICAL SCALES FOR MDD EVALUATION

The 10 clinical rating scales for MDD evaluation considered in this work are listed in Table 4. The description for each scale is provided in the last column.

Table 4. Overview of the psychometric scales.

No.	Scale	Description
1	Hamilton Depression Rating Scale (HAMD-3)	This version is not a widely recognized standard version. It may consist of 3 items selected from the original HAMD-17 for specific research or clinical contexts (Hamilton (1960)).
2	Hamilton Depression Rating Scale (HAMD-17)	This is the original version of the Hamilton Depression Rating Scale, containing 17 items (Hamilton (1960)).
3	Montgomery-Åsberg Depression Rating Scale (MADRS)	A widely used clinician-administered scale to assess the severity of depressive episodes, consisting of 10 items (Montgomery and Åsberg (1979)).
4	MADRS-S (Montgomery-Åsberg Depression Rating Scale - Self-assessed)	A self-assessment version of the MADRS, which is also usually composed of 10 items (Svanborg and Åsberg (1994)).
5	Beck Depression Inventory (BDI)	A self-report inventory created by Aaron T. Beck, designed to measure the severity of depression (Beck et al. (1961)).
6	Brief Symptom Inventory - Clinical Version (BSI-CV)	A shortened symptom self-report inventory designed to evaluate psychological symptoms and distress (Derogatis and Melisaratos (1983)).
7	Digit Symbol Test (DST)	This typically refers to the digit symbol test from the Wechsler Adult Intelligence Scale (Wechsler (1958)).
8	Digit Symbol Substitution Test (DSST)	This is a form of the digit symbol test, which may vary in different versions of the Wechsler scales (Wechsler (1958)).
9	Patient Determined Disease Steps (PDQ-D)	A self-report inventory designed to measure cognitive deficits perceived by individuals with depression.
10	Hamilton Depression Rating Scale (HAMD-6)	This is a short-form version of the Hamilton Depression Rating Scale, which typically includes 6 items selected from the original HAMD-17 to assess the severity of depressive symptoms (Hamilton (1960)).

3 THE STATE GROUPS FOR THE SIX SUBNETWORKS

The metastates are further categorized into 5 state groups according to their activity patterns, as shown in Table 5. These state groups contain brain states with activity patterns range from fully inactive to fully active.

Table 5. Categories of the metastates.

Group No.	dDMN	pDMN	vDMN	aSN	pSN	CEN
1	Major State 1	Major State 1	Major State 1	Major State 1	Major State 1	Major State 1
2	Major State 2	Major State 2	Major State 2	Major State 2	Major State 2	Major State 2
3	-	-	Minor State 1	Minor State 1	Minor State 1	Minor State 1
4	-	-	Minor State 2	-	Minor State 2	Minor State 2
5	-	-	-	-	Minor State 3	-

REFERENCES

Beck, A. T., Ward, C. H., Mendelson, M., Mock, J., and Erbaugh, J. (1961). An inventory for measuring depression. *Arch. Gen. Psychiatry* 4, 561–571

Derogatis, L. R. and Melisaratos, N. (1983). The brief symptom inventory: an introductory report. *Psychol. Med.* 13, 595–605

Hamilton, M. (1960). A rating scale for depression. *J. Neurol. Neurosurg. Psychiatry* 23, 56

Montgomery, S. A. and Åsberg, M. (1979). A new depression scale designed to be sensitive to change. *Br. J. Psychiatry* 134, 382–389

Svanborg, P. and Åsberg, M. (1994). A new self-rating scale for depression and anxiety states based on the comprehensive psychopathological rating scale. *Acta Psychiatr. Scand.* 89, 21–28

Wechsler, D. (1958). The measurement and appraisal of adult intelligence. *Acad. Med.* 33, 706
